# A Protease Inhibitor with Induction Therapy with Natural Interferon-β in Patients with HCV Genotype 1b Infection

**DOI:** 10.3390/ijms17030350

**Published:** 2016-03-09

**Authors:** Yutaka Kishida, Naohiko Imaizumi, Hirohisa Tanimura, Shinichiro Kashiwamura, Toru Kashiwagi

**Affiliations:** 1 Division of Gastroenterology and Hepatology, Department of Internal Medicine, Osaka Kaisei Hospital, Osaka City, Osaka 532-0003, Japan; nao.p@f6.dion.ne.jp (N.I.); dd9341715tam@yahoo.co.jp (H.T.); 2 Laboratory of Host Defenses Institute for Advanced Medical Science, Hyogo College of Medicine, Nishinomiya City, Hyogo 668-8501, Japan; shinchan@hyo-med.ac.jp; 3 Division of Gastroenterology and Hepatology, Department of Internal Medicine, Osaka Hospital of Japan Community Healthcare Organization, Osaka city, Osaka 553-0003, Japan; kashiwagi.toru@zeus.eonet.ne.jp

**Keywords:** chronic hepatitis C, protease inhibitor, Peg-IFN-α, ribavirin, interferon-β, induction therapy

## Abstract

The restoration of innate immune responses has potential as a novel therapeutic strategy for chronic hepatitis C (CHC). We compared the efficacy and safety of induction therapy (IT) with natural interferon-β (n-IFN-β) followed by pegylated-IFN-α/ribavirin (PR) alone (group A, *n* = 30) and IT with a protease inhibitor (PI) (simeprevir or vaniprevir)/PR (group B, *n* = 13) in CHC patients with genotype 1b and high viral loads. During IT with nIFN-β, virologic response rates in group A and group B were 10% and 8% (*p* = 0.6792) at week 4, 30% and 16% (*p* = 0.6989) at week 12 and 47% and 20% (*p* = 0.0887) at week 24 respectively. During and after the treatment with PR alone or PI/PR, virologic response rates in groups A and B were 50% and 82% (*p* = 0.01535) at week 4, 53% and 91% (*p* = 0.006745) at week 8, 57% and 91% (*p* = 0.001126) at week 12, 57% and 100% (*p* < 0.001845) at the end of the treatment and 57% and 80% (*p* < 0.005166) after treatment cessation. IT with PI/PR linked to the restoration of innate immune response was tolerated well, overcame virological breakthrough, enhanced early virologic responses, and resulted in a sustained virologic response in difficult-to-treat CHC patients. IT with PI/PR is beneficial for treating difficult-to-treat CHC patients.

## 1. Introduction

An estimated 170 million individuals worldwide are chronically infected with hepatitis C virus (HCV) and are at high risk of developing liver diseases, including liver cirrhosis and hepatocellular carcinoma [1]. Therapeutic options for the past two decades have consisted of treatments with interferon (IFN)-α and ribavirin (RBV) [2]. HCV therapeutic approaches have rapidly evolved since the approval of the first direct-acting antiviral agents (DAAs) in 2011. The development of DAAs represents a significant improvement in the treatment of HCV [3]. However, the emergence of HCV resistance associated variants (RAVs) to DAAs in the subset of patients that do not respond to DAAs represents a new issue [4]. A sustained virologic response (SVR) is used as a near-term for permanent viral eradication. However, the primary goal of therapy is not to attain SVR, but to improve the prognosis of overt liver disease and extrahepatic sequelae. Therefore, SVR is regarded as a surrogate endpoint, not a clinical endpoint [5]. These researchers described the results of a nationwide cohort study from Scotland, which included more than 80% of all chronic HCV-infected patients who underwent IFN-based therapy between 1996 and 2011. They confirmed relationships between SVR, improved quality of life, and reductions in all cause mortality. Therefore, these findings question the high cost of DAAs and whether it is reasonable for most of the HCV-infected population [6,7].

A low baseline HCV viral load has been identified as an independent predictor of SVR [8]. Viral kinetics in response to anti-HCV treatments are regarded as important factors during treatments. Successful antiviral treatments result in rapid decrease in HCV-RNA serum concentrations to undetectable levels that remain negative throughout therapy and thereafter. The faster the virus becomes undetectable during therapy, the better the chance of achieving SVR. Early responses to treatments are best determined by HCV-RNA serum levels after four and 12 weeks of therapy. A rapid virologic response (RVR) is defined as having undetectable viral levels after four weeks of therapy. RVR has a greater than 90% positive predictive value for predicting SVR. Another important viral kinetic marker during treatments is determined 12 weeks after treatments and is known as an early virologic response (EVR). A greater than 2-log10 reduction in HCV-RNA (or undetectable HCV-RNA concentrations after week 12) is defined as EVR. The negative predictive value of EVR for predicting responses was previously reported to be approximately 92% for 48 weeks of pegylated-IFN-α/ribavirin (PR) [9].

Response rates with DAA regimens are generally lower in patients who did not respond to previous treatments containing Peg-IFN-α than in treatment-naïve patients, and are markedly lower among prior null responders who are considered difficult-to-cure. Despite the enthusiasm for all-oral, interferon-free therapy, higher rates of virologic failure were seen in early evaluation of real-world data. The mechanism of relapse after therapy with DAAs is still poorly understood. New treatments are not enough to eliminate hepatitis C. Thus, more effective, tolerable, and/or tailored therapies are required for patients with a prior null response to PR therapy [10,11]. A persistent HCV infection results from inefficient innate and adaptive immunity [12]. Innate immune responses regulate adaptive immune responses via direct interactions and by means of the exchange of signals between immune cells [13,14]. HCV disturbs the activation of innate immune responses. The non-structural (NS) proteins of HCV, particularly NS3-4A, have been found to interfere with type I IFN induction pathways. IFNs are the first line of defense against viruses and act directly on viral replication and indirectly via the activation of immune responses [15]. The clearance of HCV may lead to the restoration of innate and adaptive immune responses [16,17,18]. However, many challenges are associated with achieving the solution to the induction of persistent viral suppression linked to the restoration of innate immune responses resulting in SVR. Furthermore, IFNs have no resistance to HCV which is different to DAAs. The presence of RAVs to NS5A inhibitors does not attenuate the efficacy of NS3 inhibitors or PR, and the combination of simeprevir and PR may be an alternative treatment option against Y93H RAV, other than combination therapy with DAAs [19].

**Figure 1:**
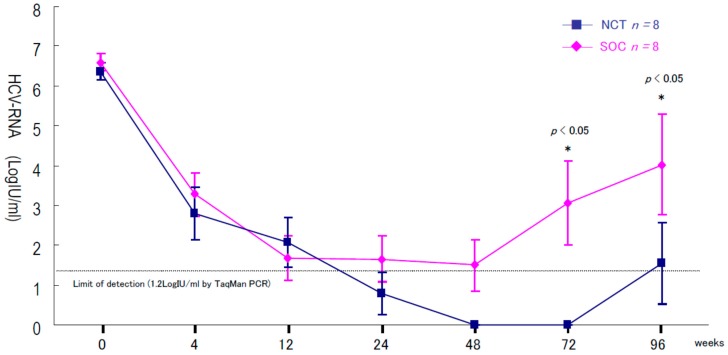
Changes in serum hepatitis C virus (HCV). RNA level during and after the NCT and the SOC in chronic hepatitis C patients with genotype 1b and high viral loads. NCT: novel combination treatment (NCT) consists of induction therapy with natural IFN-beta followed by the SOC. SOC: standard of care (SOC) consists of PegIFN-alpha 2b plus ribavirin (RBV).

**Figure 2:**
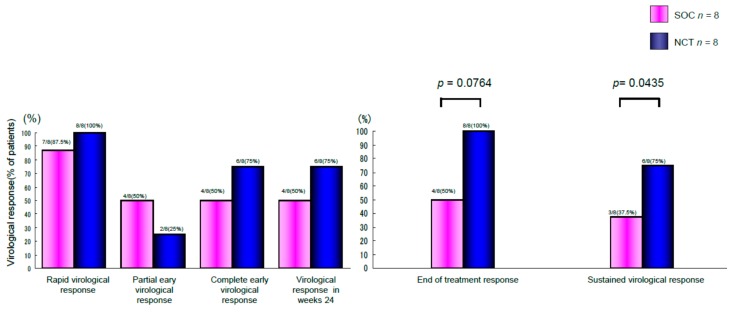
Rates of early virologic responses in the 4, 12 and 24 weeks (left panel), and end-of–treatment virologic response and sustained virologic response (right panel) in chronic hepatitis C patients with genotype 1b and high viral loads treated with the NCT or the SOC according to intention-to-treatment. NCT: novel combination treatment (NCT) consists of induction therapy with natural IFN-beta followed by the SOC. SOC: standard of care (SOC) consists of PegIFN-alpha 2b plus RBV.

The findings of our previous study showed that cyclic and periodic IFN treatment (CPIT) consisting of induction treatment with natural (n)-IFN-β and subsequent maintenance treatment with n-IFN-α induced restoration of innate immune responses, as showed by the significant decrease of CXCL-10, CXCL-8 and CCL-4, and the significant increase of interleukin (IL)-12 and IL-15 [20]. Early virologic clearance by induction therapy (IT) with n-IFN-β induced the restoration of innate immune responses linked to adaptive immune responses, which resulted in SVR. HCV viral titers significantly decreased (*p* < 0.05) from the baseline in IT followed by Peg-IFN-α plus RBV (standard of care (SOC)) and SOC after the beginning of treatment. HCV RNA levels decreased more in SOC with IT than in SOC alone (Figure 1). The rates of early virologic response differed in the initial four and 12 weeks and end treatment virologic response (ETVR) and SVR in chronic hepatitis C (CHC) patients with genotype 1 and high viral loads treated with the SOC with IT and the SOC. The rate of RVR in week four, partial EVR in week 12, complete EVR (extended RVR) in week 12, virologic response in week 24, ETVR and SVR among CHC patients with genotype 1b and high viral loads receiving the SOC and SOC with IT were 87.5% *vs.* 100%, 50% *vs.* 25%, 50% *vs.* 75%, 50% *vs.* 75%, 50% *vs.* 100% (*p* = 0.0764) and 37.5% *vs.* 75% (*p* = 0.0435), respectively. SOC with IT (*n* = 8) achieved a higher SVR rate than that of SOC (*n* = 8) in difficult-to-treat CHC patients (Figure 2). The results showed the safety of the nIFN-β treatment and supported the use of nIFN-β as a safe and alternative option. SOC with IT is more effective and have less adverse effects than SOC in difficult-to-treat CHC patients with genotype 1b and a high viral load.

The findings of our previous study [20,21,22] support the concept that viral clearance early in the course of therapy is linked to the restoration of innate and adaptive immune responses suggesting that agents providing viral suppression leading to RVR and EVR including natural (n)-IFN-β, IFN-λ, DAAs and newly developed agents may be preferable as an initial early IT. Initial viral clearance induced by a CPIT in combination with these agents may lead to the restoration of innate and adaptive immune responses, resulting in SVR in difficult-to-treat CHC patients. The restoration of innate immune responses may be a novel therapeutic strategy for chronic HCV infection [22]. In addition to the restoration of innate immune responses due to viral supression with CPIT during the initial early course of therapy, persistent virologic clearance with triple therapy consisting of protease inhibitor (PI) (simeprevir or vaniprevir) with PR is more likely to result in higher RVR, EVR, ETVR, and SVR. We previously reported that a difficult-to-cure CHC patient with genotype 1b, a high viral load, null response to previous IFN treatments, and advanced hepatic fibrosis was treated by IT with n-IFN-β followed by triple therapy with simeprevir with PR, which resulted in SVR and a sustained biochemical response (SBR) [23]. On the basis of these findings, we herein compare the efficacies and safeties of CPIT with n-IFN-β as induction therapy followed by triple therapy with PI with PR and CPIT with n-IFN-β followed by PR alone in CHC patients with genotype 1b and high viral loads. SVR rates were significantly higher among patients receiving CPIT with n-IFN-β followed by triple therapy with PI (simeprevir or vaniprevir) with PR than in those receiving CPIT followed by PR alone. CPIT with n-IFN-β followed by triple therapy with PI with PR is more effective for the treatment of difficult-to-treat CHC patients with genotype 1b and high viral loads than CPIT with n-IFN-β followed by PR alone.

## 2. Results

Table 1 summarizes the baseline demographics of the clinical, biochemical and virologic characteristics of our patients (Table 1). Group A (standard treatment with IT): thirty CHC patients were treated with CPIT with nIFN-β for 24 weeks followed by PR for 48 weeks. The outcome of previous treatments were 26 naïve, four relapsers and zero null responders in group A. Group B (PI treatment with IT): thirteen CHC patients were treated with CPIT for 24 weeks followed by PI (simeprevir for 12 weeks or vaniprevir for 24 weeks) plus PR followed by PR alone for 12–36 weeks. The outcomes of previous treatments were four naïve, five relapsers and four null responders in group B. Plasma HCV-RNA levels were 6.5 ± 0.4 Log IU/mL in group A and 6.6 ± 0.4 Log IU/mL in group B at baseline. HCV viral titers significantly decreased (*p* < 0.05) from the baseline in groups A and B after the beginning of the treatment.

**Figure 3:**
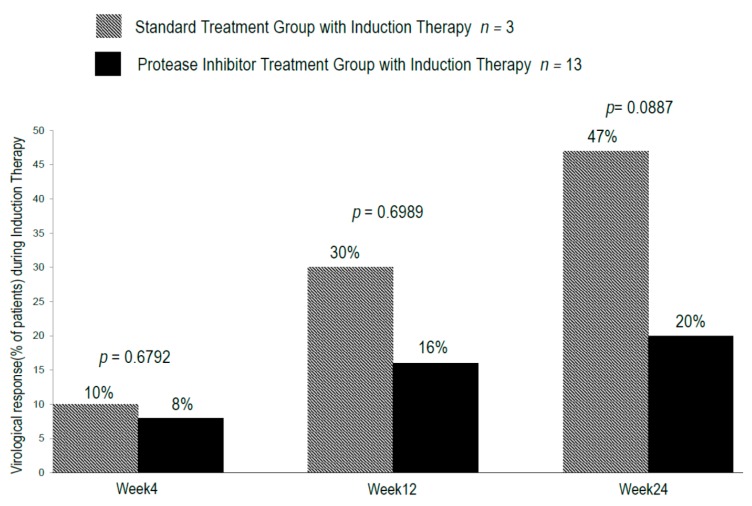
Rate of early virologic responses in the initial 4, 12 and 24 weeks in chronic hepatitis C patients with genotype 1b and high viral loads during induction therapy in standard treatment with induction therapy with n-IFN-β and protease inhibitor treatment with induction therapy with n-IFN-β. Virologic response was defined as undetectable serum level HCV.RNA (<15 IU/mL). The paired-*t* test was used to evaluate the differences of the means between two groups with a *p*-value of <0.05 considered significant.

Figure 3 illustrates the distribution of virologic responses observed during IT (Figure 3).

**Figure 4:**
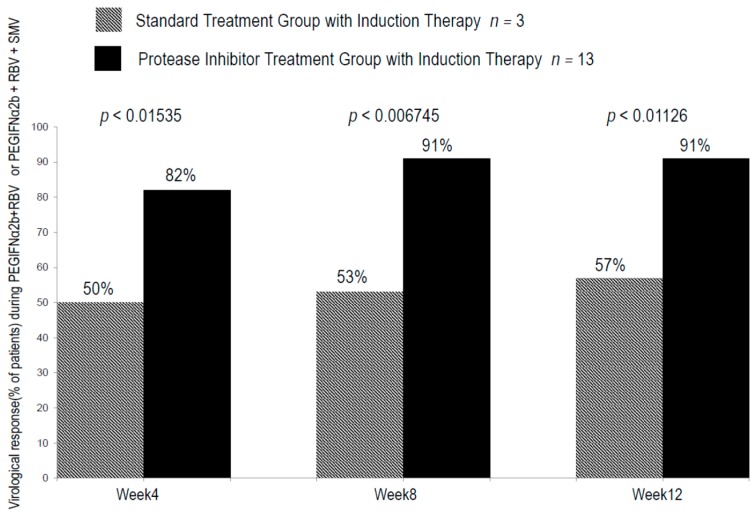
Rate of early virologic responses in the initial 4, 8 and 12 weeks in chronic hepatitis C patients with genotype 1b and high viral loads during standard treatment with induction therapy with nIFN-β and protease inhibitor treatment with induction therapy with nIFN-β. Virologic response was defined as undetectable serum level HCV.RNA (<15 IU/mL). The paired-t test was used to evaluate the differences of the means between two groups with a *p*-value of <0.05 considered significant.

**Figure 5:**
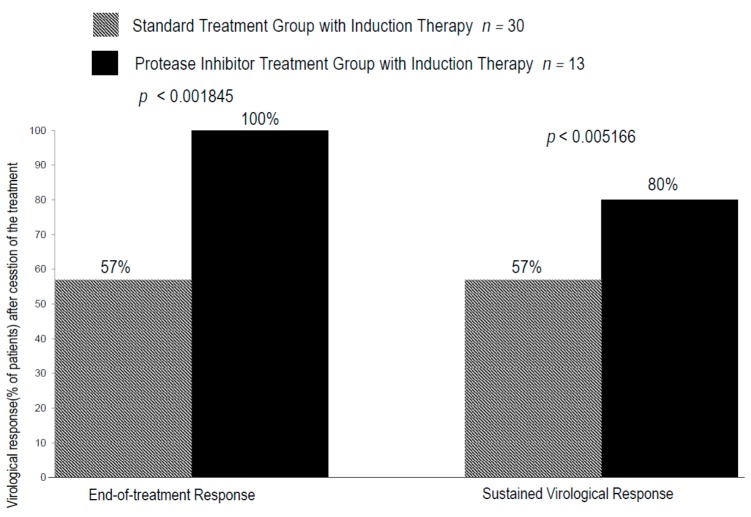
Rate of virologic responses at the end-of treatment and rate of sustained virologic response in chronic hepatitis C patients with genotype 1b and high viral loads in standard treatment with induction therapy with nIFN-β and protease inhibitor treatment with induction therapy with nIFN-β. Virologic response was defined as undetectable serum level HCV.RNA (<15 IU/mL). The paired-*t* test was used to evaluate the differences of the means between two groups with a *p*-value of <0.05 considered significant.

During IT with CPIT, virologic response rates in groups A and B were 10% and 8% (*p* = 0.6792) at week four (RVR), 30% and 16% (*p* = 0.6989) (EVR) at week 12 and 47% and 20% (*p* = 0.0887) at week 24 (at the end of IT) respectively. During and after the treatment with PR alone or PI with PR, virologic response rates in groups A and B were 50% and 82% (*p* = 0.01535) at week 4, 53% and 91% (*p* = 0.006745) at week 8, 57% and 91% (*p* = 0.001126) at week 12, 57% and 100% (*p* < 0.001845) at the end of the treatment and 57% and 80% (*p* < 0.005166) 12 weeks after the cessation of the treatments (Figure 4 and Figure 5).

Two patients in group B showed virologic relapse. One of the two patients, who showed the HCV variant with a mutation at NS3 aa position D168 that was resistant to NS3A PI and the major type of the IL 28B genotype (rs8099917, rs1188122, rs8103142), relapsed four weeks after the treatment with PI with PR, was discontinued. Another patient, who showed the HCV variant with a mutation at NS3A aa position D168A/V that was resistant to NS3A PI and the HCV variant with a mutation at NS5A aa position Y93H that was resistant to NS5A and the hetero-type of the IL28B genotype (rs8099917, rs1188122, rs8103142) relapsed four weeks after the treatment with PI with PR was discontinued. No patient exhibited virological breakthrough. During IT with CPIT, virologic response rates were higher in group A than in group B. However, during the treatment with PI with PR or PR after the cessation of IT virologic response rates were significantly higher in group B than in group A.

The overall safety profile was similar in the two groups. Serum levels of alanine aminotransferase (ALT) in groups A and B (65.2 ± 51.6 *vs.* 44.3 ± 23.7 IU/L at baseline) decreased to 29.3 ± 18.2 (*p* = 0.0010) in group A and to 29.0 ± 21.2 (*p* = 0.2262) in group B 12 weeks after the cessation of the treatment. Platelets levels in the peripheral blood of CHC patients with genotype 1b and high viral loads in groups A and B (18.2 ± 6.8 *vs.* 16.0 ± 5.0 × 10^4^/micro-l at baseline) decreased during IT and improved significantly earlier in group A than in group B (16.9 ± 4.4 *vs.* 10.9 ± 2.3 × 10^4^/micro-l, (*p* < 0.026)) at the end of the treatment and 19.2 ± 4.1 *vs.* 11.9 ± 2.8 × 10^4^/micro-l, (*p* < 0.009) 12 weeks after the cessation of the treatment.

Hemoglobin (Hb) levels in the peripheral blood of CHC patients with genotype 1b and high viral loads in groups A and B (13.3 ± 1.3 *vs.* 13.0 ± 1.8 g/dL at baseline) were markedly lower in group B than in group A (9.8 ± 1.2 *vs.* 11.5 ± 1.5 g/dL, (*p* < 0.011)) at the end of the treatment and 11.4 ± 1.7 *vs.* 12.2 ± 1.7 g/dL (*p* = 0.289) 12 weeks after treatment cessation.

Safety: The most common adverse events (AEs) observed were myalgia, anemia, general fatigue, anorexia and alopecia, and most AEs were mild. Treatments were tolerated well.

## 3. Discussion

IFN-free, DAA-based antiviral strategies and an understanding of their future clinical use in patients with CHC have markedly improved. High SVR rates are achievable with IFN free, DAA-based regimens in patients with CHC. In clinical trials with new IFN-free, DAA-based therapies, treatment failure occurred in 5%–7% of patients on average. Most treatment failures were post-treatment relapses and, and at the time of failure, these patients harbored viral populations that were resistant to >1 of the DAAs administered [24,25,26,27,28,29]. Patients who fail to eradicate HCV on DAA-containing regimens are likely to select viral populations that are resistant to the DAAs administered. Retreatment strategies must be assessed in patients who still harbor resistant viruses at the time of retreatment. HCV variants resistant to NS3A PI and to non-nucleoside inhibitors of the HCV RNA polymerase are currently selected in patients who do not respond to therapies based on these drugs. However, they decrease progressively following treatment interruption and are no longer detectable within 12–16 months of the treatment. In contrast, this is particularly important in patients exposed to NS5A inhibitors, because these variants appear to persist as dominant species for many months to years, maybe for life, after treatment failure [4]. HCV resistance, particularly resistance to NS5A inhibitors, is emerging as a novel issue in patients who fail to achieve SVR with IFN-free, DAA-based therapy. Sequential treatments with non-curative prior DAA therapies may generate complex resistant variants that limit future treatment options. For example, NS5B resistance has not been observed in treatment-naïve patients failing sofosbuvir (SOF)-containing regimens; however, SOF-associated NS5B resistance variants have emerged when patients who relapsed after SOF-containing regimens were retreated with SOF-Ledipasvir [30]. Although HCV RAVs are not archived with hepatitis B virus, they may persist for months to years, thereby making retreatment decisions more complex. It has not yet been established whether these patients treated with IFN-free, DAA-based therapies will have long-term benefit from the clearance of HCV infection despite recent improvements in the efficacy of these therapies. However, these medicines are still very expensive. When medicines are introduced, their use will be severely restricted because of their high cost.

Patients have achieved viral clearance with IFN-based treatments [31]. IFN-based treatments are considered to be more useful in the treatment of CHC patients with the major type of the IL28B genotype than IFN-free DAA treatments. The mechanism underlying the IFN susceptibility of Y93H RAV currently remains unclear. Previous studies showed that Y93H RAV was less frequently detected in patients with the unfavorable IL28B genotype non-TT (rs8099917). In other words, Y93H RAV may be more appropriate for replicating in the liver of IL28B TT patients than in those with non-TT. The most prominent difference in the liver environment between IL28B TT and non-TT is the hepatic expression of IFN stimulated genes. The hepatic expression levels of IFN stimulated genes are higher in non-TT patients, which may reflect the basal activation of the intrinsic IFN system. One possible hypothesis is that the Y93 wild type has the ability to prevail over RAVs with high levels of intrinsic IFN in IL28B non-TT patients. The concept of this hypothesis is consistent with the previous finding that Y93 RAV is more susceptible to externally administered IFN [19]. Furthermore, RBV is a useful adjunct of therapy, particularly in the most difficult-to-cure patients. RBV appears to be useful for increasing the rates of SVR. However, the use of RBV is, more frequent associated with side effects. Viral clearance may be impaired as a result of immune defects. HCV directly impairs immune function by interfering with IFN production and signaling. Therefore, the suppression of HCV by DAAs may restore innate immune function, thereby augmenting the benefits of therapy [32,33,34]. The benefits of IT with CPIT in enhancing RVR and EVR rates may be relevant in treatment strategies involving the combination of IT with CPIT with nIFN-β followed by PR with DAAs in difficult-to treat CHC patients. In our previous study [23], we demonstrated that a difficult-to-cure patient with HCV genotype 1b, who was a prior null responder to IFN treatments and had chronic active hepatitis with advanced fibrosis was successfully treated by IT with CPIT with nIFN-β followed by PI (simeprevir) with PR, and that these treatments were tolerated well with only mild AEs being noted. The present study showed that the virologic responses rates of IT with CPIT with nIFN-β followed by PI with PR were significantly higher than those of IT with CPIT with nIFN-β followed by PR alone in patients infected with HCV genotype 1b and high viral loads. During IT with CPIT, virologic response rates in group B were lower than those in group A. These lower virologic response rates may be attributed to the outcomes of previous treatments; group A had 0 null responders (0/30 (0.0%)), whereas group B had 4 (4/13 (30.7%)). However, during and after the treatment with PR alone or PI with PR, virologic response rates were significantly higher in group B than in group A. CPIT with nIFN-β followed by PI with PR was tolerated well, enhanced RVR, EVR, ETVR and SVR rates in difficult-to-treat CHC patients with genotype 1b and high viral loads, and only had mild AEs. Higher virologic response rates highlight the benefit of PI with PR with IT with nIFN-β in CHC patients with genotype 1b and high viral loads. Early virologic clearance by CPIT with nIFN-β for 24 weeks before the beginning of PI with PR induced the restoration of innate immune responses linked to adaptive immune responses resulting in SVR and SBR. SVR rates were significantly higher in CHC patients with genotype 1b and high viral loads among patients receiving CPIT followed by PI with PR than in those receiving CPIT with nIFN-β followed by PR alone. CPIT followed by PI with PR was more effective for treating difficult-to-treat CHC patients with genotype 1b and high viral loads than CPIT with nIFN-β followed by PR alone. Persistent HCV clearance continued in our patients with a reduction in HCV-RNA levels due to IT followed by triple therapy. These results suggest that IT associated with reductions in HCV-RNA levels before the beginning of triple therapy with PI with PR may be used to treat difficult-to-cure CHC patients with genotype 1b and high viral loads.

### Two Patients in Group B Showed Virologic Relapse

One of the two patients, who had a HCV variant with a mutation at NS3A aa position D168 that was resistant to NS3A PI and the major type of the IL28B genotype (rs8099917, rs11881222, rs8103142), relapsed four weeks after the treatment with PI with PR was discontinued. The other patient, who showed the HCV double variant with a mutation at NS3A aa position D168 that was resistant to NS3A PI and the HCV variant with a mutation at NS5A aa position Y93H that was resistant to NS5A and the hetero type of the IL28B genotype (rs8099917, rs1188122, rs8133142), relapsed four weeks after the treatment with PI with PR was discontinued. These patients were considered to be virologic relapsers (DAA failure) and difficult-to-cure. The treatment of patients with RAVs using DAAs who fail to eradicate HCV on DAA-containing regimens remains unresolved. Retreatment with SOF with IFN and/or RBV in previous failure may result in SVR 12 based on initial studies. One potential reason for this result is that SOF is in a different therapeutic class and has been demonstrated to have a very high barrier to resistance [35]. Virologic relapsers may be successfully treated with IT with CPIT with nIFN-β followed by DAAs, such as the NS5B polymerase inhibitor, with PR as a retreatment strategy. The results of this preliminary study indicate the need for a prospective assessment of the possible role of viral reductions with IT followed by triple therapy in difficult-to-treat CHC patients.

## 4. Patients and Methods

### 4.1. Study Design

CPIT: Patients were treated with six cycles (24 weeks) of CPIT. One cycle of CPIT consisted of induction treatment with nIFN-β (Feron^®^, Toray, Tokyo, Japan) at 3–6 MU/day, intravenously injected by a drip infusion in 100 mL of saline solution, daily for 2 weeks followed by maintenance treatment with nIFN-α (Sumiferon^®^, Sumitomo, Osaka, Japan) at 6 MU/day, self-subcutaneously injected, once every two days for 2 weeks.

Group A: standard treatment with IT (*n* = 30): CPIT for 24 weeks as IT followed by Peg-IFN-α-2b ((Pegintron^®^, MSD, Whitehouse Station, NJ, USA) 100–120 μg/day, once a week, percutaneous injection) and ribavirin (RBV) ((Rebetol^®^, MSD, Whitehouse Station, NJ, USA) 200–1000 mg/day, peroral, daily) for 48 weeks (a total of 72 weeks).

Group B: PI treatment with IT (*n* = 13): CPIT for 24 weeks as IT followed by PI (simeprevir or vaniprevir) plus Peg-IFN-α-2b (Pegintron^®^, MSD, Whitehouse Station, NJ, USA, 100–120 μg/day, once a week, percutaneous injection) and RBV (Rebetol^®^, MSD, Whitehouse Station, NJ, USA, 200–1000 mg/day, peroral, daily) for 24 weeks (a total of 48 weeks). Ten CHC patients were treated with simeprevir (Sovriad^®^, Janssen, Titusville, NJ, USA, 100 mg/day per os, daily) for 12 weeks. Three CHC patients were treated with vaniprevir (Vanihep^®^, MSD, Whitehouse Station, NJ, USA, 400 mg/day, peroral, daily) for 24 weeks.

Dose modifications: Dose modifications to Peg-IFN-α 2b and RBV were performed for AEs and laboratory abnormalities. Adjustments to the daily dose of RBV were performed in gradual stepwise decrements of 200 mg. RBV was withheld in patients who had a Hb level of less than 8.5 g/dL. Patient adherence to therapy was assessed via recording injections and the doses of Peg-IFN-α 2b, and RBV at each visit according to the patient’s detailed statements, and via documentation of the drug dispensed.

We compared the efficacies and safeties between group A (*n* = 30) and group B (*n* = 13) in CHC patients with genotype 1b and high viral loads. Patients had undergone liver biopsy within 6 months prior to treatment initiation. In one patient in group B, hepatic fibrosis was assessed with FibroScan (Fibroscan^®^, Echosens SA, Paris, France) and showed 5.3 kPa (F0–1). All patients were monitored using clinical and biochemical assessments, virologic responses were determined before and every 1 to 4 weeks during the 48–72-week treatment period, and were followed for at least an additional 24 weeks after treatment cessation. HCV-RNA serum levels were determined using the quantitative TaqMan real time PCR (Roche Diagnostic Systems, Tokyo, Japan; sensitivity < 15 IU/mL) (Table 1). Direct sequencing of the NS3 and NS5A regions of HCV-RNA was successfully generated for two patients in group B. The IL28B genotype had been determined by the rs8099917, rs1188122, and rs8103142 single-nucleotide polymorphism for the polymerase chain reaction (PCR) amplification and sequencing of patients in group B.

Written informed consent was obtained from all study patients. The study was conducted in accordance with the Declaration of Helsinki, and the protocol was approved by the Ethics Committee in Japan.

### 4.2. Assessments and Efficacy Endpoints

The primary efficacy endpoint was SVR defined as undetectable HCV-RNA serum levels 12–24 weeks after treatment completion. Secondary endpoints included RVR defined as undetectable HCV-RNA serum levels at the end of 4 weeks of treatment, and EVR defined as undetectable HCV-RNA serum levels at the end of 12 weeks of treatment.

### 4.3. Measurements to Improve Treatment Adherence

Full adherence to all drugs is associated with high SVR rates. In contrast, suboptimal exposure to therapy is associated with virologic breakthrough or post-treatment relapse and the emergence of RAVs, especially during the early phase of treatment. Prior to the initiation of antiviral therapy, patients were informed of the schedule and side effects to be expected during the treatment. Patients were instructed on preventive and therapeutic measures to ameliorate these side effects. Regular follow-up visits were scheduled for treatment progression and the management of eventual side effects to be discussed.

### 4.4. Assessments of Safety

AEs were monitored every 1–4 weeks during and after the end of the treatment. Laboratory (hematology and biochemistry) tests, vital signs, symptoms of fatigue, flu-like symptoms, electrocardiogram assessments, and physical examinations were performed at screening and thereafter at regular intervals throughout the treatment. Peg-IFN-α-2b was reduced from 60–120 μg to 50–100 μg/day for the management of AEs or laboratory abnormalities that had reached the predetermined thresholds of severity. The RBV dose was reduced from 1000 to 200 mg per day in order to manage AEs or laboratory abnormalities that had reached the predetermined thresholds of severity. If AEs were resolved or improved, a return to initial dosing levels was permitted.

### 4.5. Exclusion Criteria

The following were considered as exclusion criteria:

Refusal by women of child -bearing age or by sexually active patients to use a safe contraceptive, pregnancy or breast-feeding, cirrhosis with signs of decompensated liver diseases, coronary heart diseases, the presence of overt psychiatric diseases, active alcohol or drug abuse, uncontrolled diabetes mellitus, uncontrolled hypertension, uncontrolled retinopathy, autoimmune disorders, or any other unstable medical condition not due to liver disease. Frequent causes of chronic liver diseases were excluded.

### 4.6. Statistical Analysis

Data were expressed as the mean±standard deviation and a paired *t*-test was used to evaluate the differences in the means between groups, with a *p* value of <0.05 being considered significant.

## 5. Conclusions

The findings of our previous study showed that CPIT consisting of induction treatment with natural (n)-IFN-β and subsequent maintenance treatment with n-IFN-α could prevent viral breakthrough and achieved RVR and EVR, and induced restoration of innate immune responses, as shown by the significant decrease of CXCL-10, CXCL-8 and CCL-4, and the significant increase of IL-12 and IL-15 in CHC [20]. Early virologic clearance by IT with n-IFN-β induced the restoration of innate immune responses linked to adaptive immune responses, which resulted in SVR. In our previous study, the higher SVR rates in patients with genotype 1b HCV infection among patients receiving IT with nIFN-β followed by PR compared with SVR rates in those receiving PR alone were revealed [21]. In addition to the restoration of innate immune responses due to viral suppression with CPIT during the initial early course of therapy, persistent virologic clearance with triple therapy consisting of PI (simeprevir or vaniprevir) with PR is more likely to result in higher RVR, EVR, ETVR, and SVR. We previously reported that a difficult-to-cure CHC patient with genotype 1b, a high viral load, null response to previous IFN treatments, and advanced hepatic fibrosis was treated by IT with n-IFN-β followed by triple therapy with simeprevir with PR, which resulted in SVR and a SBR [23]. On the basis of these findings, we herein compare the efficacies and safeties of CPIT with n-IFN-β as induction therapy followed by triple therapy with PI with PR and CPIT with nIFN-β followed by PR alone in CHC patients with genotype 1b and high viral loads. Viral suppression linked to the restoration of innate immune responses with IT with n-IFN-β followed by PI plus Peg-IFN-α-2b and RBV was tolerated well without discontinuation, overcame viral breakthrough and induced persistent viral clearance, leading to an enhanced early virologic response, and resulted in SVR 12 and SBR in difficult-to-treat CHC patients with genotype 1b and high viral loads. SVR rates were significantly higher among patients receiving CPIT with n-IFN-β followed by triple therapy with PI (simeprevir or vaniprevir) with PR than in those receiving CPIT followed by PR alone. It also effectively eradicated HCV infection and caused mild AEs in difficult-to-treat CHC patients. 

In conclusion, CPIT with n-IFN-β as IT followed by triple therapy with PI plus PR is more effective for the treatment of difficult-to-treat CHC patients with genotype 1b and high viral loads than IT with nIFN-β followed by PR alone.

## Figures and Tables

**Table 1:** Standard Treatment; treatment with pegylated (Peg) interferon (IFN)-α2b and ribavirin (RBV). Protease inhibitor treatment, treatment with protease inhibitor (simeprevir or vaniprevir) plus Peg-IFN-α 2b and RBV; Induction therapy, treatment with cyclic and periodic IFN treatment which consists of induction treatment with natural IFN-β and maintenance treatment with natural IFN-α. ALT, alanine aminotransferase; HCV, hepatitis C virus; Hb, hemoglobin in the peripheral blood.

Characterristics	Standard Treatment with Induction Therapy (*n* = 30)	Protease Inhibitor Treatment with Induction Therapy (*n* = 13)
Sex (Male/Female)	10/20	2/11
Age (years), mean ± SD	58.9 ± 10.1	64.0 ± 8.7
Weight (kg), mean ± SD	58.2 ± 10.1	58.7 ± 10.4
Body mass index (kg/m^2^), mean ± SD	23.2 ± 3.9	24.0 ± 3.3
ALT (IU/L), mean ± SD	65.2 ± 51.6	44.3 ± 23.7
HCV RNA (Log IU/mL), mean ± SD	6.5 ± 0.4	6.6 ± 0.4
Liver histology (Stage; F), *n* (%)		
F0	2 (7)	1 (8)
F1	15 (50)	4 (31)
F2	7 (23)	3 (23)
F3–4	3 (10)	3 (23)
Missing	3 (10)	2 (15)
Hb (g/dL), mean ± SD	13.3 ± 1.3	13.0 ± 1.8
Platelets (10^4^/μL)	18.2 ± 6.8	16.0 ± 5.0
Outcome of previous treatment naïve/relapse/null responder 10/20	26/4/0	4/5/4
